# Online listing data and their interaction with market dynamics: evidence from Singapore during COVID-19

**DOI:** 10.1186/s40537-023-00786-5

**Published:** 2023-06-11

**Authors:** Jieun Lee, Kwan Ok Lee

**Affiliations:** 1 Department of Real Estate, National University of Singapore, Singapore, Singapore; 2 Department of Real Estate, NUS Business School , National University of Singapore, Singapore, Singapore

**Keywords:** Online listing, Natural language processing, Advertising keywords, COVID-19, PropTech

## Abstract

With the emergence of Property Technology, online listing data have drawn increasing interest in the field of real estate–related big data research. Scraped from the online platforms for property search and marketing, these data reflect real-time information on housing supply and potential demand before actual transaction data are released. This paper analyzes the interactions between the keywords of online home listings and actual market dynamics. To do so, we link the listing data from the major online platform in Singapore with the universal transaction data of resale public housing. We consider the COVID-19 outbreak as a natural shock that brought a significant change to work modes and mobility and, in turn, consumer preference changes for home purchases. Using the Difference-in-Difference approach, we first find that housing units with a higher floor level and more rooms have experienced a significant increase in transaction prices while close proximity to public transportation and the central business district (CBD) led to a reduction in the price premium after COVID-19. Our text analysis results, using the natural language processing, suggest that the online listing keywords have consistently captured these trends and provide qualitative insights (e.g. view becoming increasingly popular) that could not be uncovered from the conventional database. Relevant keywords reveal trends earlier than transaction-based data, or at least in a timely manner. We demonstrate that big data analytics could effectively be applied to emerging social science research such as online listing research and provide useful information to forecast future market trends and household demand.

## Introduction

While the real estate sector has traditionally lacked the digitalization process, the emergence of big data and the Internet of Things has recently led to a boom in technological advancement, called Property Technology (PropTech). Online listing platforms, which are one of the most significant PropTech initiatives, provide a unique context where consumers collect online information before viewing offline commodities which feature very heterogenous attributes in terms of interior and exterior design, size, material, etc. [14, 71]. These platforms have been widely used by potential homebuyers and real estate marketers. By obtaining useful information, consumers are able to save time and money by picking and choosing only the homes they are interested in. And to better attract the attention of consumers, real estate marketers carefully choose keywords to advertise homes.

With the emergence of PropTech, online listing data have drawn increasing interest in the field of real estate–related big data research. Scraped from the online platforms, these data manifest real-time information on housing supply and potential demand as the main goal of online listings is to attract consumers’ interests with effective advertising strategies [37]. On the other hand, some developed countries like Singapore publicly provide universal data of actual housing transactions with various property characteristics. Based on the transaction data, we could understand consumers’ willingness to pay for housing units with certain features [63] and accompanied market dynamics. If there are significant shocks to consumer preference changes for homes, consumers would reveal these changes in their transaction behaviors [11, 41, 42] while real estate marketers would also reflect these changes in their online listing advertisement. Therefore, there should be a dynamic interrelationship between online listing data and actual market trends.

In this paper, we attempt to analyze whether and how quickly keywords for online home listings reflect actual trends in consumers’ housing preferences. We use the COVID-19 outbreak as a natural shock to the local housing market in Singapore. As the COVID-19 pandemic has limited public mobility and transformed work arrangements, and in turn changed consumer preferences for their homes, it provides a good opportunity to explore how quickly advertising keywords have captured changing preferences revealed in actual transaction outcomes. For example, we would expect increasing popularity for more spacious homes with a nice view while accessibility to public transportation would be a less important factor for home-buying decisions in the post-COVID-19 period. Analyzing the online listing data is also useful for a more comprehensive understanding of consumer preference changes. For example, keywords may highlight housing features, such as views and maintenance, which are not captured in the standard transaction database but important for homebuying decisions.

We use the data of over 96,000 resales transactions of public housing provided by Singapore’s Housing and Development Board (HDB) for the period of 2018 to 2021, together with more than 76,000 online listings (after preprocessing the original 350,000 listings), from the major online listing company, PropertyGuru, which has 84% of the market share regarding agent subscription revenue in Singapore [24]. To understand housing features that become more or less popular after the COVID-19 shock from actual transactions, we use the hedonic pricing model. In doing so, the Difference-in-Difference (DID) modelling approach is applied to account for temporal variation (before vs. after COVID-19) as well as variations in key housing features (i.e. floor level, floor size, proximity to a subway station and the central business district [CBD]). Then, we explore the keyword trend of online listings by performing word vectorization involving word embedding, based on keyword topics related to housing features that are shown to have a significant change in their popularity in the housing market. We also compare the temporal trends between housing price premiums and advertising keywords, using the event study specifications.

Broadly, this research sits in the literature on data-driven decision sciences with a focus on online marketing (e.g. [1, 21, 66]. In particular, we consider advertising keywords as a means to reflect a dynamic change in consumer preferences revealed in actual market transactions. A focus on property listings provides us with the excellent opportunity to examine how online advertising interacts with actual market trends, which could also be outcomes (i.e. transactions) of advertising. Real estate agents play an important role in this process as they are both the online marketer and sales person on behalf of landowners. They spend a significant amount of time and effort to make listings more attractive as they get commission generally based on the amount they sell [38, 51, 80]. Our analyses attempt to test how precisely and quickly advertising keywords capture changes in consumer preferences and whether these keywords provide additional qualitative information that the actual transaction database fails to reveal (e.g. increasing preference for an unblock view).

Our paper provides insights into the expanded scope and value of big data analytics in emerging research on online listings. Several researchers ventured into using structured and unstructured text data from the property listing portals to study diverse topics on real estate, from predicting housing prices [9, 19, 20] and estimating investability [67], to evaluating the effectiveness of advertising [55]. In the real estate field, scholars used online listing descriptions to measure the uniqueness of properties and focused on housing prices as an outcome [54, 68, 69]. Different from these papers, we focus on market dynamics and investigate how advertising keywords respond to consumer preference changes regarding the specific housing feature. By performing advanced methods such as word vectorization, we are able to capture the temporal trends of advertising keywords. These trends, in addition to online keyword search volume or consumer sentiment from major news articles [12, 45, 75], could help forecast housing markets. Our approach is also more interdisciplinary because we perform economic analyses of actual transaction data and then link them to text analyses. We believe that one advantage is to have a better contextual understanding of critical topics rather than solely relying on text pattern recognition algorithms.

Finally, our paper adds big data–based evidence to the ongoing discussions on how consumer preferences for homes have been reshaped by increasing work-from-home (WFH) arrangements after the COVID-19 outbreak. A series of research suggested that the COVID-19 pandemic has influenced housing demand especially through the substantial increase in WFH along with reduced mobility because of restriction measures and infection concerns (e.g. [16, 27, 50, 81]. Researchers reported that residential demand has increased in neighborhoods with lower density and crowdedness [10, 48] and in housing units with more space for the home office and terrace [15, 16, 81]. Based on existing evidence, we encompass both physical and locational features of housing units for our analysis. We account for the dynamic trends of advertising keywords and accompanied qualitative information that cannot be captured by studies relying on survey or transaction data. Our research also features Singapore’s unique context for residential choices within one city and focuses on multifamily housing units in the high-density environment. Using the resale public housing data makes it easier to disentangle the price premium changes associated with specific features like a higher floor level or proximity to a subway station, because units are quite homogeneous in terms of design and community facilities [43]. We attempt to test the external validity of existing evidence from other countries.

The rest of this paper is organized as follows. The next section provides brief scholarly and institutional backgrounds for our research followed by the section that describes data and methodology. Next, the main results of this research are presented with the COVID-19 effects on changes in housing price premiums with respect to different housing features, and the exploratory text analysis shows how keywords reflect actual market dynamics. Finally, we conclude and provide implications.

## Background

### COVID-19 outbreak, consumer preference change, and willingness to pay

The COVID-19 pandemic has brought a significant change in consumers’ housing preferences, especially with the dramatic increase in work-from-home (WFH) arrangements along with infection concerns [16, 27, 44, 50, 81]. Also, national lockdowns and social distancing measures have required people to conduct daily activities at home [39]. Research has reported a shift in consumer demand for larger space, both in the home’s interior and exterior [15]. At the same time, the COVID-19 shock has influenced preferences for residential locations. While homes in densely populated areas in cities had been valued for their access to amenities such as shopping malls and railway stations as well as proximity to workplaces, these amenities became less attractive to potential homebuyers because of the perceived risk for infection [10, 48]. As a result, a decline in demand was reported to be strongest in high-density neighborhoods and central cities [48].

In accordance with the theory of revealed preference [64], we could observe consumer choices and their willingness to pay as an indicator of their preferences for the specific feature of a product or service [11, 41, 42]. When consumers significantly change their preferences for the product due to an external shock such as the COVID-19 pandemic, they would reevaluate their priority or willingness to pay. For example, many consumers changed their behaviors after COVID-19, particularly toward health- and safety-related goods and online services [33, 36]. We could investigate these consumers’ preference changes based on their transaction outcomes in the market [63]. In the midst of the COVID-19 pandemic, many researchers paid attention to multidimensional changes in revealed consumer preferences from actual transactions (e.g. [11, 41, 42]).

In a similar fashion, some research relied on the actual property transaction outcomes to examine consumers’ preference changes for homes. For example, Boesel et al. [15] use the CoreLogic Single-Family Rent Index and demonstrated that homes with larger interior living space and larger lots experienced a higher increase in their housing prices. The interpretation of this finding is associated with increasing consumer preferences for separate spaces for working individuals who need a home office and school-age children who take in-home classes. Others utilize the survey to directly ask consumer about their preference changes for homes. For example, using the questionnaire with 632 respondents in Tehran, Zarrabi et al. [81] report that consumer demand has increased for homes with natural light, visibility, interior spaces with better acoustics, and open or semi-open space. We add big data–based evidence to this ongoing research on consumers’ preference changes for homes after COVID-19. Our analyses encompass changes revealed from the universal transaction data with specific housing features and changes inferred from the advertising keywords of online listing data. We compare the extent of consistencies and temporal trends of findings from two different datasets.

### Consumer preference change, advertising, and online property listings

Based on the Engel Kollat Blackwell (EKB) model, consumer decisions involve five steps including problem/need recognition, information search, evaluation of alternatives to meet the need, purchase decision, and post-purchase behavior [28]. If we apply this theoretical concept to our research topic, consumers recognize needs driven by COVID-19 (e.g. more home space), search for information in the online platform, and make housing transactions. On the other hand, to attract consumer’s attentions and purchase intentions, advertising reflects consumer behaviors and their preferences rather than simple descriptions of the project [6, 53]. Hence, real estate marketers would observe changes in consumer needs during and after transaction processes and reflect them in their online advertising. These processes are dynamically interrelated with each other because customers would evaluate alternative housing options based on online listing information before making their homebuying decisions.

To illustrate practical backgrounds of online property listings, the National Association of Realtors [52] reported that 95% of homebuyers use online platforms to search for homes, and over half of them end up purchasing the housing units they found on these platforms. The first step most homebuyers across all age groups take during their home purchase process is searching the internet for available properties [52]. Keywords attract people’s attention to connect sellers’ target audience with a detailed webpage of each listing. When the keywords effectively catch the attention of target customers, they could bring more potential buyers to the product’s webpage and increase the probability of successful sales [29, 62, 79].

The existing evidence suggests that online listing descriptions affect the transaction prices of advertised housing units. Haag et al. [34] suggested that while both objectively verifiable and subjective keywords have a significant price effect, some keywords falling in the former category (e.g. good location) have a negative association with selling prices potentially because they are hype. Goodwin et al. [30] argued that the price effect of online keywords is more significant in heterogeneous, detached housing markets. Sing and Zou [69] suggested that online listing keywords could play an important role in the price premium for advertised housing units if they deliver essential information and provide easy-to-read and mood-independent descriptions. As the scholarly interest has risen in utilizing structured and unstructured text data from the property listing portals, non-real estate researchers have studied technical aspects of these data [9, 19, 20, 55, 67] We attempt to contribute to this thin literature by expanding the scope of big data analytics and providing comprehensive implications to multiple disciplines of decision sciences, marketing, and real estate.

### The Singapore context

Singapore’s government has responded to the COVID-19 pandemic with a range of measures such as strict border controls; contact tracing; home isolation with the closure of schools, universities, and workplaces; social distancing; and allowing only essential businesses, such as grocery stores and banks, to open. The government imposed a circuit breaker, a stay-at-home order that mandates the closing of non-essential businesses and transitioning to home-based learning, on 3 April in 2020. After the end of the circuit breaker on June 1, three phases of gradual reopening were imposed. In Phase 1 (2–18 June, 2020), offices re-opened, but work-from-home was mandated to the maximum extent. In Phase 2 (19 June–27 December, 2020), work-from-home was a default arrangement, and the group size of gatherings was restricted to five people. In Phase 3 (28 December 2020–July 2021), the government changed safety measures based on domestic COVID-19 cases, ranging from work-from-home as a default to a requirement of only 50% or 75% of workers in offices.

These strict measures lasting over one year have significantly affected not only the national economy but also individuals’ lives in Singapore. Work-from-home (WFH) and home-based learning became a default lifestyle for most households while their social gatherings were largely limited. These have significantly decreased public mobility and changed household housing demand in Singapore. Survey results show that most respondents consider WFH as a longer-term trend and they are willing to trade commuting time and other preferences for housing with more space and a better view [26, 70]. In addition, anecdotal evidence suggests that as the whole family spent most of their time at home, more households wanted larger spaces [47]. Some households decided to move away from the central area to the less expensive, suburban areas to larger housing units.

Given that the COVID-19 outbreak and associated measures mainly affected locals’ lives and changed their housing demand, our analyses focus on online listings and public housing transactions in Singapore. Public housing in Singapore is developed and sold by a government agency called the Housing and Development Board (HDB). According to the 2021 Singapore Statistics, 78.3% of Singapore resident households resided in public housing. While foreign investors can purchase private housing and drive its market trends, public housing units can only be purchased by Singapore citizens or permanent residents. These units can be purchased at a subsidized price when directly buying from the HDB, but after a 5-year minimum occupation period, homeowners of public housing are allowed to sell their units on the open market without price control. The online listing and transaction database used for all analyses are for resale public housing. Hence, the changes in keywords for online listings and price premiums observed from actual transactions can capture the trends in the local public housing market.

## Data and methodology

### Data

We use two datasets for real estate properties that could potentially be considered big data in the real estate field [7, 17, 18, 22, 32, 76, 78, 83]. The first is a dataset on universal resale transactions of public housing from January 2018 to December 2021 in Singapore. The data are collected by the Housing Development Board (HDB) as the HDB administers public housing transactions and all resale transactions should be reported to the HDB. Therefore, this data has full coverage of all resale market transactions and include transaction price, floor level, floor area in square meters, the number of rooms, location, and age of each residential building. Although the sample size could be a lot bigger if we account for all historical transactions, we used the balanced panel (2 years both before and after COVID-19) so our final sample size is 96,028. We mainly used the data to estimate the change in consumer preferences revealed through their willingness to pay for different housing features after the COVID-19 outbreak.

The second data source is propertyguru.com.sg, the biggest online house listing platform, which has 84% of the market share regarding agent subscription revenue in Singapore. We collected home listings at 10 distinct time points over the period from September 2019 to July 2021 using a python-based web-scraping tool. While we started with the initial data containing about 350,000 listings, we only kept listings for standard resale public housing (multi-family, high-rise housing) to focus on local homebuyers’ preference changes driven more by user demand rather than investment demand. After preprocessing and cleaning duplicate data, our final sample contains more than 76,000 sales listings with various information including unit characteristics (i.e. the number of rooms, floor area, floor level category [high/middle/low]), location characteristics (i.e. latitude and longitude, neighborhood town), building age, and the asking price. The data also include the free text descriptions used to advertise each home listing, which is important for our analyses.

Table 1 provides descriptive statistics of the resale transactions of public housing used for our empirical analyses. In our first analysis of actual housing demand change, our sample covers the data from January 2018 to December 2021 to test longer-term price trends associated with key housing features. Compared to transactions before the COVID-19 outbreak (column 2), housing units traded after COVID-19 (column 3) show higher transaction prices. Results also suggest that units with more rooms were transacted more frequently after COVID-19 while the average floor area of transacted units did not change significantly. In terms of public transport accessibility, units closer to the nearest subway station were transacted less after COVID-19. These may suggest potential changes in transaction trends in resale public housing markets.

**Table 1:** Summary statistics of public housing resale transaction data

	(1) Whole sample of transactions		(2) Pre-COVID19: Jan 2018–Dec 2019		(3) Post-COVID19: Jan 2020–Dec 2021	
Number of observations	96,028		43,602		52,426	
Variable	Mean	Std. Dev	Mean	Std. Dev	Mean	Std. Dev
Unit characteristics						
Real price (S$)	460,672	159,633	434,537	155,012	482,409	160,161
Price per sqm	4,740.91	1,290.56	4473.01	1,218.38	4,963.73	1,306.47
% 10^th^ or higher floor	38.25	48.60	37.73	48.47	38.69	48.70
% four or more rooms	75.32	43.11	74.26	43.72	76.20	42.58
Floor area (sqm)	97.85	24.15	97.73	24.37	97.96	23.96
Building age	26.42	13.70	27.97	12.75	25.12	14.32
Location characteristics						
Distance to the closest subway station (m)	781.98	448.94	763.73	446.11	797.15	450.72
% near subway (within 400 m)	19.18	39.37	20.20	40.15	18.33	38.69
Distance to CBD (km)	10.42	4.49	10.58	4.61	10.28	4.39
% near CBD (within 2 km)	3.82	19.16	3.85	19.24	3.79	19.09
Distance to the closest park	1,316.28	685.87	1,331.73	685.68	1,303.43	685.77
Distance to the closest mall	626.10	360.07	629.71	364.86	623.09	356.02
Distance to the closest primary school	598.01	720.37	591.93	719.74	603.07	720.86
Temporal characteristics						
% Post-COVID-19	54.59	49.79	0.00	0.00	100.00	0.00

All monetary values are in real terms adjusted using monthly CPI. S$1 = US$0.995 as of December 2019

### Methodology

#### Empirical model to examine COVID-19 effects on actual housing price premiums

To explore how housing price premiums have changed with respect to key housing features after COVID-19 based on actual resale transactions, we employ difference in differences (DID) as follows:

1 $$y_{it} = \alpha + \delta D_{i} + \gamma T_{i} + \beta D_{i} T_{i} + \theta X_{i} + \varphi F_{tj} + e_{i}$$

where ${y}_{it}$ is the transaction price per square meter of the housing unit *i* in month* t*, ${D}_{i}$ is the binary treatment indicator of whether unit *i* has a certain housing feature (i.e. 10th or higher floor, four or more rooms including the living room, within 400 m to a subway station, and within 2 km to the CBD or not), and ${T}_{i}$ is the binary indicator of whether unit *i* was transacted before or after the COVID-19 outbreak. The coefficient of the interaction term of ${D}_{i}$ and ${T}_{i}$,$\beta$ reflects the change in the price premium of a housing feature of our interest after the COVID-19 outbreak. ${X}_{i}$ is a control vector of unit- and location-specific characteristics. Unit characteristics include the floor level and area of the unit, building age, and their square terms. Location characteristics include the distances to the closest subway station, park, primary school, and mall and the distance to the CBD. ${F}_{tj}$ is the vector of stringent fixed effect terms regarding both geographical (street) and temporal (year-month) variations to increase the probability that the treatment effect is as exogenous as possible. In our DID analyses, our treatment group is housing units with specific housing features (e.g. high-floor units) while our comparison group is units without these features (e.g. low-floor units) in the database of public housing resale transactions. Because we include the stringent fixed effect terms regarding both geographical (street) and temporal (year-month) in our DID regressions, actual comparison group should be units that are located on the same street and transacted in the same month and year but do not have a specific housing feature.

#### Text analysis to explore online advertising trends for homes after COVID-19

To understand whether and how online advertising captures the trends from actual housing markets shown above, we focus on description headline texts obtained from online listings and perform natural language processing (NLP) such as word cloud, unigram, and bigram analyses. Research has frequently used NLP to categorize unstructured texts into certain patterns and generalize the common trends from the texts [4, 40]. Real estate literature on online advertising also used NLP to understand soft features which could be obtained only from unstructured text data, in addition to hard attributes of properties [2, 31, 35, 54, 57].

Following the other papers which conducted topic modeling [20, 56, 77], we performed preprocessing before our text analysis using the *re* module [74]. The detailed process is as follows. First, we removed html tags, web links (which starts with http, https) and symbols such as apostrophes, brackets, and line feeds (\n). Second, we converted all of the alphabets into lower case. Third, we removed standard stopwords sourced from the nltk.corpus package [13] and some custom stopwords to ensure our corpus be filled with meaningful words. Our custom stopwords were ‘exclusive listing’, ‘unit’, ‘sale’, ‘listing’, ‘call’, ‘now’, and ‘enquire’ because these words cannot add meaning in our sample for our analysis. Fourth, we performed lemmatization with WordNetLemmatizer. After tokenizing all of the words with the word_tokenize function of the nltk library, we replaced some tokens with a customized dictionary to transform some abbreviations to standard words (e.g. paring ‘yrs’ with ‘years’, ‘bdr’ with ‘bedroom’, ‘rm’ with ‘room’, and ‘mins’ with ‘minutes’).

Then, we depict word clouds before and after COVID-19 for the exploratory purpose. After the brief overview of keyword trends, we perform N-gram analyses with unigrams and bigrams. With the pre-processed texts, we rank the top 1000 most frequently appearing keywords in each year and calculate the share of the frequency of each keyword (${p}_{it}$) out of the total frequency sum of each year. Then, we calculate and visualize the percent changes of the share of each keyword in the years 2020 and 2021 compared to 2019 to understand their trends after the COVID-19 outbreak. We also perform the bigram text analysis in the same manner but with a pair of words. In doing so, we categorize keywords into different dimensions such as view-related, space-related, and location-related, and display results by each category. We determine these categories to be related with housing features that we focus on in our DID model introduced above. For example, we compare results for view- and space-related keywords with DID results for the floor level and size, respectively, while associating location-related keywords with the proximity to the subway station and CBD.

#### Empirics to compare the temporal trends of markets and advertising keywords

In addition to the above DID model, we investigate the quarterly movement of price premiums compared to the first quarter of 2019 with an event study identification, which was commonly used to address the parallel trends issues for DID approaches [43, 46]. This allows us to relax our identification with multiple time windows and observe the dynamic changes in price premiums for each housing feature. By overlaying these changes with the temporal trends of relevant keywords from online listings, we attempt to find out how quickly the online listing keywords respond to, or even lead, the changing trends in housing markets.

To quantify the temporal trends of the extent to which listings feature keywords associated with main housing features derived from the above event study, we process word vectorization and convert keywords in the same topics to get the same numerical representations [59, 65]. For this process, we apply four different topics for keyword vectors (i.e. view, rooms/space, public transport, central location), indicating whether each listing contains related keywords or not. In comparing the quarterly trends of actual transaction outcomes (i.e. price premiums) and advertising keywords (i.e. share of listings featuring the keywords), we note that housing transaction data are usually available one month later. This means that there may be a slight lag time for the real estate marketers to adjust keywords to market trends.

## Results and discussions

### COVID-19’s effects on housing demand change based on actual transactions

Table 2 displays estimates of the Difference-in-Difference (DID) model presented in Eq. (1) with price per square meter as a dependent variable. The average transaction prices increased after the COVID-19 outbreak in Singapore. Other countries have shown heterogeneous housing market outcomes in the post-COVID-19 period. For example, studies in China have reported a reduction or minor change in housing transaction volume and prices, especially in the regions where infection rates were high and the government maintained anti-speculation policies [23, 58, 72, 82]. However, in many other countries, housing prices have significantly increased due to low interest rates, a desire for more space as people work from home, and accumulated savings during the lockdown [60]. In the case of Singapore, the main reason for the price boom was the limited supply because of delayed construction coupled with strong investment demand from overseas because of the stability of the Singapore housing market as well as domestic demand rising from higher liquidity [73].

**Table 2:** COVID-19 Effects on price premiums associated with actual housing features (Difference-in-difference regression results)

Dependent variable: resale price per square meter								
	(1) 10th + floor		(2) 4 + rooms (3 + bedrooms with a living room)		(3) Within 400 m to subway		(4) Within 2 km to CBD	
Post-COVID-19	453.06 (16.26)	***	449.89 (15.70)	***	477.06 (15.19)	***	467.52 (14.95)	***
Post-COVID-19 × 10th or higher floor	35.13 (5.60)	***						
10th or higher floor	303.57 (4.18)	***						
Post-COVID-19 × four or more rooms			18.10 (6.40)	***				
Four or more rooms			− 121.74 (5.02)	***				
Post-COVID-19 × near subway					− 61.78 (6.85)	***		
Near subway					256.00 (6.01)	***		
Post-COVID-19 × near CBD							− 76.14 (16.62)	***
Near CBD							74.04 (26.66)	***
Location characteristics	Y		Y		Y		Y	
Unit characteristics	Y		Y		Y		Y	
Year & Month FE	Y		Y		Y		Y	
Street name FE	Y		Y		Y		Y	
N	96,027		96,027		96,027		96,027	
Adjusted-R^2^	0.90		0.91		0.91		0.91	

^*^p < 0.1; **p < 0.05; ***p < 0.01. Robust standard errors in parentheses

For public housing in Singapore, the living room is included in the number of rooms. So we essentially capture whether the unit has three or more bedrooms

Our main interest lies in the extent to which the average price premium has changed with respect to key housing features, including the floor level, spaciousness, proximity to subway stations, and proximity to the CBD, after the COVID-19 outbreak. We first find that the units located on a higher floor level have experienced an increase in their transaction price by $35.13 per square meter after COVID-19 (column 1, Table 2). Similarly, the transaction price for units with three or more rooms has significantly increased after COVID-19 and the premium change appears to be $18.10 per square meter (column 2). Existing evidence suggests that semi-open spaces, like balconies in apartment units, have become more important for residents who have been forced to stay at home during COVID-19 [8, 49]. Hence, a potential explanation for our result is that mobility restrictions and staying home longer during the lockdown period and beyond have increased demand for open air and better views from homes located on a higher floor level. At the same time, more activities done at home, such as WFH and home-based learning, have likely increased families’ desire for more separate spaces [15, 39].

On the other hand, while the proximity to subway stations generally has a positive effect on price changes, units located within 400 m of the nearest subway station have experienced a reduction of $61.78 per square meter after COVID-19 (column 3). Similarly, units located within 2 km of the CBD enjoyed higher premiums before the pandemic, but these premiums have almost disappeared afterward (column 4). This reduction in price premiums associated with locational attributes appears to come mainly from trading these attributes for other features. Our additional analysis (not shown) suggests that units with decreased price premiums after COVID-19 are likely to have fewer rooms or be on a lower floor level while being located closer to subway stations or the CBD. In other words, homebuyers have transferred some portion of their premium payment from locational advantages to higher floors and more rooms after COVID-19.

These results are consistent with anecdotal evidence that lifestyle changes such as WFH as a default mode and Singapore’s strict mobility restrictions reduced the desire to live closer to public transportation and the CBD [70]. Singapore primarily consists of high-density urban areas. However, our findings suggest that changes in housing market trends are similar to other large countries like the US, where many households moved to suburban areas and home prices near transit fell in the post-COVID period [3, 15, 48, 61].

### How online listing keywords capture consumer preferences and market trends

**Fig. 1:**
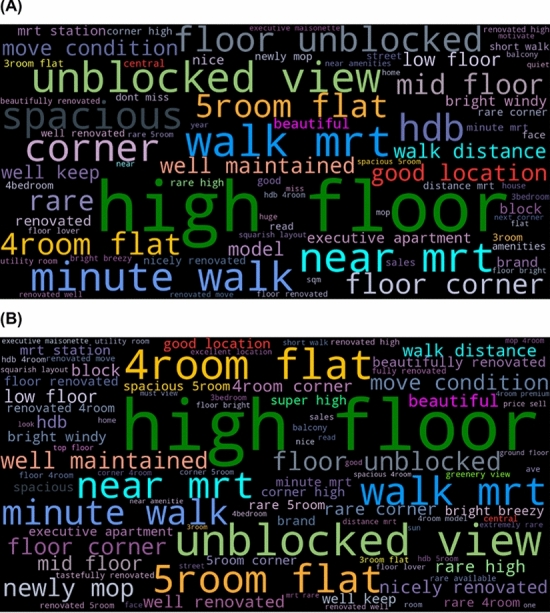
Word Clouds with Advertising Keywords before (**A**) and after (**B**) COVID-19

We now test whether the advertising keywords on the online listing platform captured the housing market trend and how these keywords uncover changes in other dimensions that we were unable to find with conventional transaction data. We begin with the word-cloud analysis that allowed us to explore frequently appearing groups of words before and after the COVID-19 outbreak (Fig. 1). In line with the classic hedonic model results, the number of rooms, floor level (e.g. high/mid/low floor), and proximity to public transportation have been frequently advertised. For example, keywords indicating larger units such as “4room flat” and “5room flat” were used more frequently after COVID-19 while keywords such as “near mrt”, “walk mrt”, “minute walk”, and “good location” were less used. A new finding is that qualitative features like “unblock view” and “well maintained” tend to be advertised more frequently in online listings in the post-COVID-19 period.

**Fig. 2:**
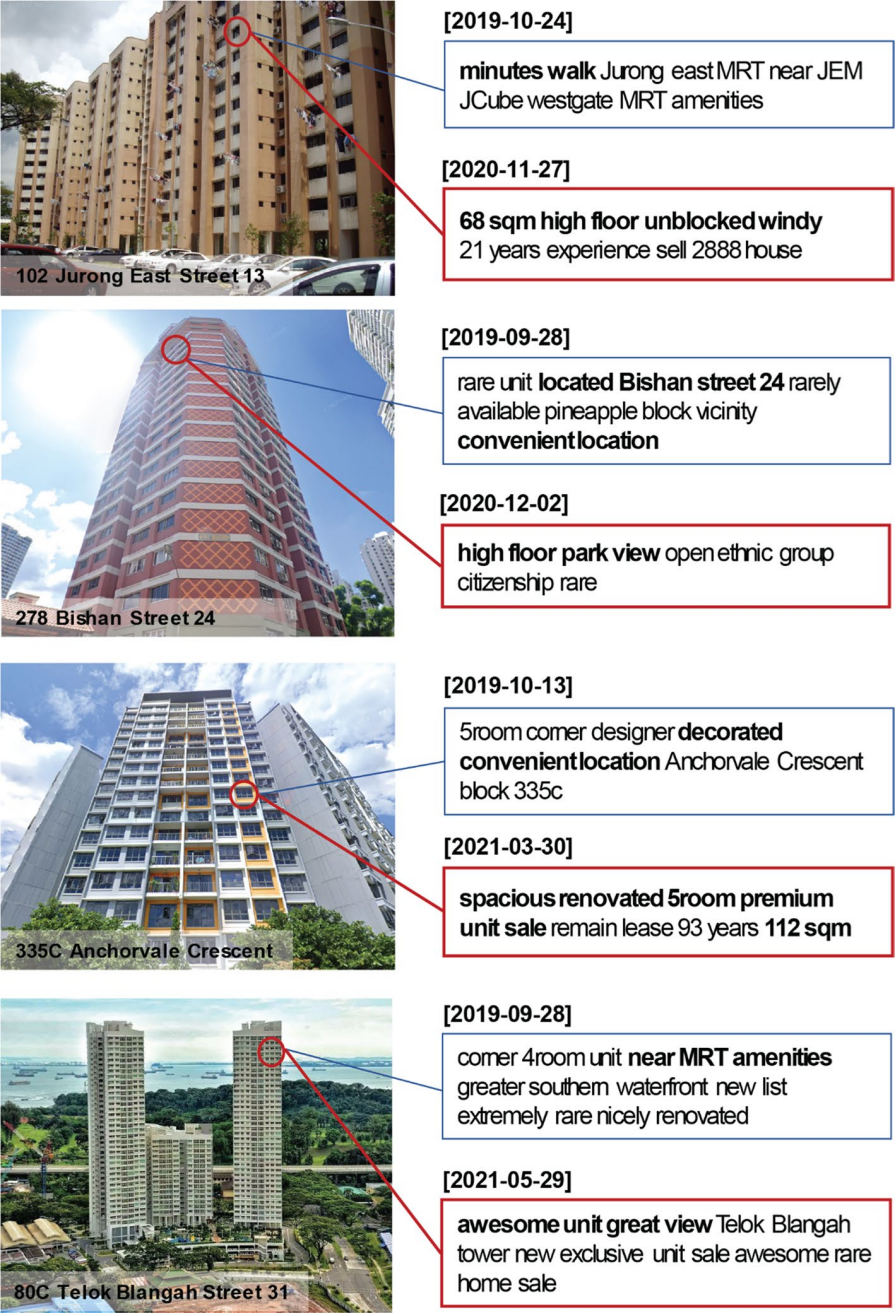
Example Cases for Keywords Changes for Units with Homogeneous Attributes

We could also find how the units that are almost identical in terms of address, floor level, and floor area are advertised with different keywords before and after the COVID-19 outbreak. As shown in Fig. 2, while units in the same building, on the same floor, and with the same size were advertised for their convenient location and short distance to subway (i.e. Mass Rapid Transit, MRT) stations in 2019, they were advertised for their high floor with unblocked view and the large number of rooms in the post-COVID-19 period. Motivated by this anecdotal evidence, we carried out a paired sample t-test for statistical inferential analyses. Our pairs share the same address, floor level, and floor area, and we define the following as null hypotheses: H_0_: The mean frequency of keywords per each listing related with four topics (i.e. view, space, transport, and central location) does not significantly differ in pre- versus post-COVID-19 periods. Results (not shown but available upon request) strongly validate (with p < 0.01, one tail) the alternative hypotheses for all four topics. For example, the mean frequency of view-related keywords is significantly higher for the post-COVID-19 period compared with the pre-COVID-19 period (0.475 vs. 0.443; t = 9.11), while the transport-related keywords appear a lot less frequently after COVID-19 (0.275 vs. 0.322, t = − 14.02) for paired units with the same size, location, and floor level.

**Fig. 3:**
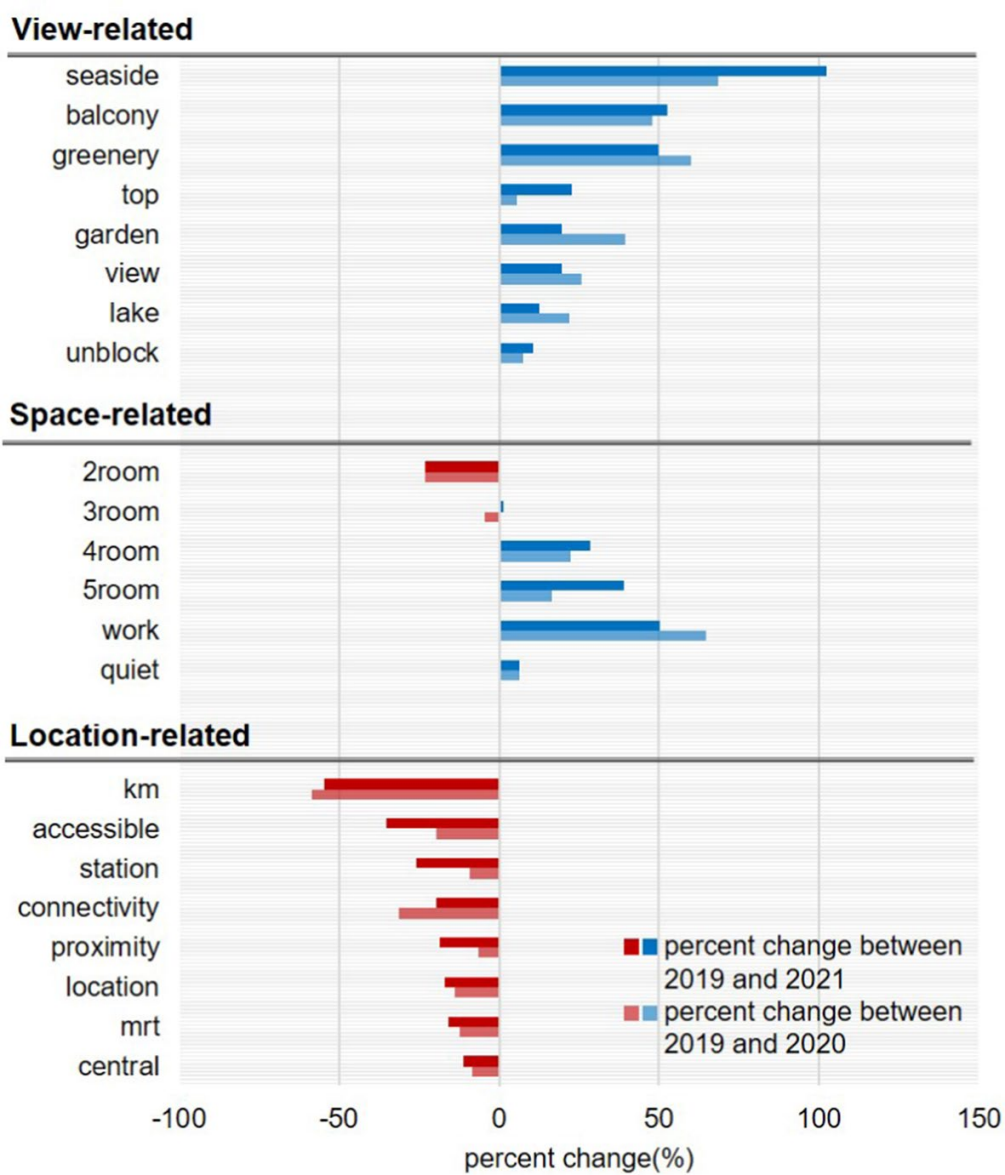
Unigram Analysis Results

**Fig. 4:**
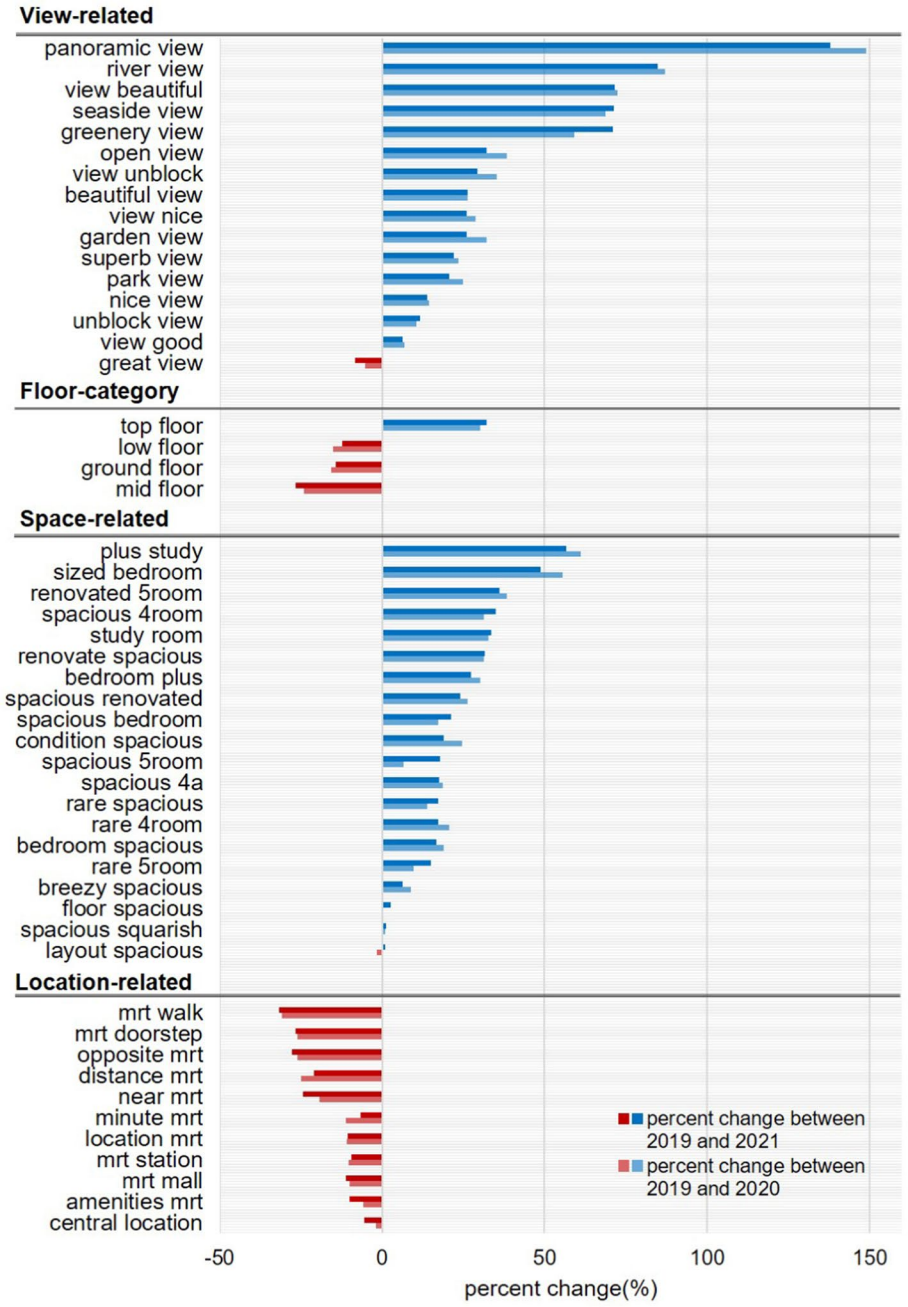
Bigram Analysis Results

To provide more concrete analysis results, we next perform unigram and bigram text analyses and report the percent change in the relative frequency of advertising keywords between pre- and post-COVID-19 periods. First, both Figs. 3 and 4 suggest that view-related keywords appeared in online listings more frequently after the COVID-19 outbreak. For example, a single word of “view” and dual words directly indicating a good view such as “panoramic view”, “river view”, “view (is) beautiful”, “greenery view”, and “open view” have significantly increased their appearance in listings in 2020 and 2021 compared to 2019. In addition, less direct words such as “top”, “unblock”, and “balcony” also increasingly appeared in listing headlines after the COVID-19 outbreak (Fig. 3). Because units on the higher floor level are more likely to feature a better view than lower-level units, this result is consistent with the previous finding that higher-level units experienced increased price premiums after COVID-19 (Table 2, column 1). Figure 4 further shows that the probability of being advertised with “top floor” increased by more than 30 percent after COVID-19, whereas the keywords of “low”, “ground”, and “mid-floor” appeared much less frequently.

Next, in terms of space-related features, unigram analysis results show that 4- and 5-rooms became popular keywords for units in 2020 and 2021 compared to 2019, while having 2 rooms is advertised a lot less (Fig. 3). In addition, keywords suggesting a better living environment, such as “work” and “quiet”, appear more frequently after COVID-19. Bigram analysis results similarly show that keywords indicating more separate spaces within the housing unit with better housing quality—such as “plus study”, “renovated 5room”, and “spacious 4room”—appeared a lot more frequently in the post-COVID-19 periods (Fig. 4). These keywords directly reflect the increasing popularity of public housing units with more rooms shown in Table 2 (column 2). As mentioned above, we believe that this is mainly caused by the increasing WFH trend and higher demand for homes to accommodate diverse activities like home-schooling or teleworking.

Finally, both unigram and bigram analyses demonstrate that location-related words appear less often in the headline of online listings after the COVID-19 outbreak. For example, single words presenting the distance or certain location such as “km”, “connectivity”, “proximity”, “station”, “mrt”, and “central”, appear less frequently after COVID-19 (Fig. 3). We also discover that dual words presenting public transportation accessibility, such as “mrt walk”, “opposite mrt”, “distance (to) mrt”, and “mrt station”, appear less frequently (Fig. 4). This downward trend is directly linked to a significant reduction in the price premium for proximity to public transportation and the CBD after COVID-19 shown in Table 2 (columns 3 and 4). While highlighting the convenient and accessible locations used to be critical for online advertising, real estate agents have likely changed their strategies to adapt to new market trends.

These results suggest that trends in online listing keywords are very consistent with our earlier findings based on actual transactions. In addition, keywords from the online listings allow us to understand qualitative features associated with housing market changes that cannot be uncovered by standard hedonic pricing models using the transaction data. For example, although we find increased premiums for units on higher floor levels in Table 2, we do not fully understand the reasons. Our text analyses add insights that households have a growing desire for an open and unobstructed view, which is closely associated with the floor level, after the COVID-19 outbreak.

### Temporal trends of price premiums and advertising keywords

**Fig. 5:**
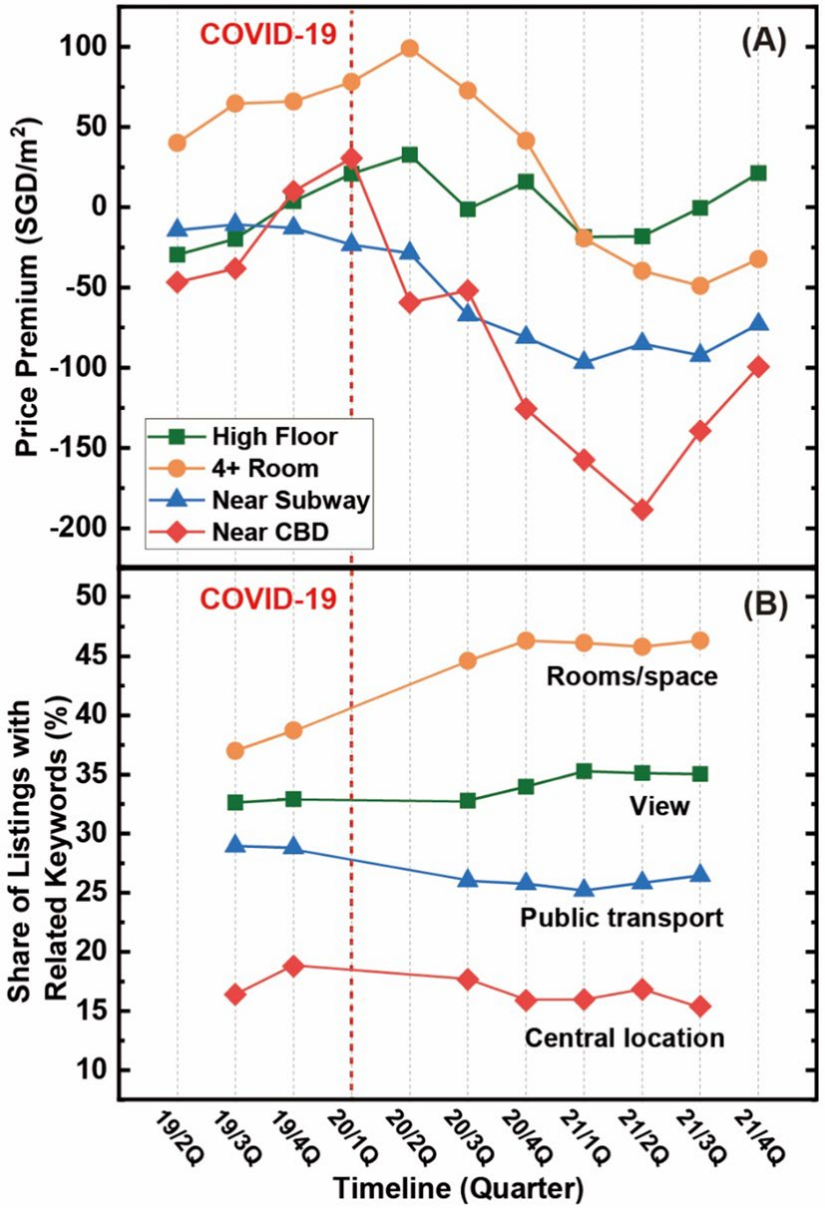
Trends for Price Premiums (**A**) and Listings with Related Keywords (**B**). Note 1: Panel A shows the trend of price premiums with the event study analyses focusing on how the price premium for each feature changed in each quarter compared to the 1st quarter of 2019 as a baseline. Panel B shows the trend of the share of listings advertised with each keyword category at each data collection point. Note 2: The keywords we used for each category in Panel B are as follows: (1) ‘view’ keywords include river view, seaside view, greenery view, open view, beautiful view, garden view, superb view, park view, nice view, unblock view, panoramic view, view beautiful, view good, great view, high floor, balcony; (2) ‘rooms/space’ keywords include plus study, sized bedroom, study room, bedroom plus, spacious bedroom, bedroom spacious, 4 full bedroom, 5 full bedroom, 5room, 4room, 5bedroom, 4bedroom, 3bedroom, 5i, 5a, 4a; (3) ‘public transport’ keywords include mrt, lrt, bus, train, station; (4) ‘central location’ keywords include central, city centre, cbd, central district names(e.g. raffles, orchard), and ‘location’. Note 3: For the online listings, we collected data at 10 distinct time points as follows: October 28, 2019; September 29, 2020; November 2, 2020; December 4, 2020; January 14, 2021; February 21 2021; March 26, 2021; March 29, 2021; May 27, 2021; and July 19, 2021

How quickly do these keywords capture changes in actual transaction outcomes? As shown in Panel A of Fig. 5, the price premiums for the units with more rooms and on a higher floor level have significantly increased from the 2nd quarter of 2020. We believe that this is related to strict COVID-19 measures in Singapore, because the 2nd and 3rd quarters of 2020 were during and right after the lockdown (7 April–1 June, 2020). Anecdotal evidence from several real estate agents suggests that many customers were looking for new units with more space and a better view even during the lockdown period. These home-seekers have likely acted quickly, and changes in their preferences would have been reflected in changes in price premiums for associated housing features.

At the same time, the real estate marketers should have learned consumer preference changes from their interaction with customers as well as the housing transaction database and reflected them when they put up the online advertising. In this regard, we find that the shares of online listings highlighted with space- and view-related keywords increased significantly in the 3rd and 4th quarter of 2020 when WFH became a default working mode, respectively (Panel B, Fig. 5). We note that we do not have the listing data for the 2nd quarter so our observation can only begin from the 3rd quarter. Considering the slightly lagged release of transaction data and a substantially smaller number of transactions in the 2nd quarter of 2020 (3225 in the 2nd quarter vs. 7365 in the 3rd quarter), we believe that real estate marketers responded to market trends in a timely manner.

For the location characteristics, the price premiums for units that are proximate to a subway station and the CBD show significantly decreasing trends from the 3^rd^ and 4^th^ quarter of 2020, respectively (Panel A, Fig. 5). The reduction on the share of listings highlighted with public transport–related keywords became significant from the 3rd quarter of 2020 (Panel B), which coincides with the time when the price premium for the proximity to the closest subway station became negative. Similarly, central-location keywords show a substantial reduction in their presence in the listing headlines right after the COVID-19 outbreak (Panel B). The price premium reduction for units located within 2 KM of the CBD was highest among all housing features, and advertising keywords well reflected these market trends. Overall, we claim these results are suggestive evidence that online listing keywords capture dynamic changes in housing market trends earlier than the transaction outcomes, or at least in a timely manner.

## Conclusions

The dynamic interrelationship between online listing keywords and consumer behaviors manifested in actual transactions has important implications on housing markets, especially when there is a strong shock and dynamic changes in the markets. Our analyses linking the keywords of online listings with housing price premiums from the transaction database provide a promising opportunity to estimate such interrelationships more accurately. Using the difference-in-difference model and the database of resale public housing transactions in Singapore, we first find that housing units on a higher floor level and with more rooms have experienced a significant increase in transaction prices while proximity to public transport and the CBD leads to the lower prices after COVID-19. Hence, our results confirm the external validity beyond the lower-density, Western context of a shifting trend of housing demand from smaller units in the urban area to larger units in the suburban area (e.g. [3, 48, 61]). We believe that these similarities mainly arise from the fact that both Singapore and these countries experienced significant transitions to work-from-home (WFH) trends after COVID-19.

Our text analysis results suggest that the online listing keywords have quickly captured the above trends based on the listing data from the major online platform in Singapore. Consistent with the results on transaction price premiums, both unigram and bigram analyses suggest a significant increase in the frequency of keywords related to view and more space while showing a decrease in the frequency of keywords highlighting the proximity to public transportation and a central location. These keywords show similar trends earlier than actual transaction outcomes, or at least in a timely manner. Moreover, our text analysis results provide useful qualitative information that could not be learned from the conventional transaction database. For example, our evidence that view-related keywords increasingly appear on online listing headlines after COVID-19 and WFH transitions further explains why units with a higher floor level have become more expensive among post-COVID-19 transactions.

Our contributions to big data research communities are two folds. On the one hand, we demonstrate that big data analytics could effectively be applied to emerging social science research such as online listing research. By doing so, we try to contribute to expanding the scholarly discussions surrounding big data, from big data mining and relevant methodologies to the broadened scope and value of big data analytics. For example, we used word embedding to analyze keywords of online listing data, and analysis results help provide a more comprehensive understanding of housing market dynamics. On the other hand, our study connects changes in revealed consumer preferences from the housing transaction data with changes in advertising keywords for online property listings. This has implications beyond real estate research because it suggests how online marketing adapts to quickly reflect actual consumer preferences in highly dynamic circumstances such as the COVID-19 pandemic, especially for the products without popular brands like housing [5].

Our analysis findings also provide practical implications. They suggest that the online listing data for housing could be utilized to forecast changes in household housing demand and evaluate the dynamic trend of real estate markets. These data could easily be accessed on a real-time basis so they become publicly available a lot earlier than administrative data that government agencies collect and report several quarters or years later. Therefore, the cycle of capturing market trends could be much shorter, and the real estate industry could adjust in a timely manner. For example, real estate developers and marketers could be better tailored to the preferences of consumers. In particular, the capacity of real estate marketers to quickly capture and highlight these features is likely to play an important role in their performance in a dynamically changing environment. Likewise, urban planners and policymakers could utilize the data to understand changes in citizens’ housing demand, especially for public housing. In countries like Singapore where the majority of households reside in public housing or public policies drive private housing markets, the government’s plan has a strong impact on the size, location, and density of future housing development.

Our work has limitations, which could spark future research. First, our online listing data were collected at only 10 distinct time points and the sample size was not very large. Ideally, we would have wanted to retain more frequent data for both pre- and post-COVID-19 periods over a longer horizon so that we can conduct more rigorous empirical analyses and apply more advanced big data techniques. Next, our analyses do not fully account for the underlying mechanisms through which actual transaction outcomes and online marketing behaviors influence each other. Although this is beyond the scope of our current research, it will be extremely interesting to examine how real estate marketers acquire information of market trends and strategize their advertising as well as how consumer sentiment and preferences for different housing features can be reshaped by online advertising. Future research may consider involving other big data, such as social media data, to study this. Finally, our analyses relied on Singapore, which features the unique housing market in a city state. Future research would benefit greatly from more cross-country comparisons using big data analytics. Also, because COVID-19 measures varied significantly by country, more comparative analyses would be useful to generalize empirical results on the housing market dynamics and advertising keywords after the COVID-19 outbreak.

## Data Availability

The datasets generated and analyzed during the current study are available from the corresponding author on reasonable request.

## Abbreviations

CBD: Central Business District
HDB: Housing and Development Board
DID: Difference-in-difference
WFH: Work-from-home
NAR: National Association of Realtors
CPI: Consumer Price Index
NLP: Natural Language Processing
MRT: Mass Rapid Transit
